# Characterization of heterotrophic growth and sesquiterpene production by *Rhodobacter sphaeroides* on a defined medium

**DOI:** 10.1007/s10295-019-02201-6

**Published:** 2019-06-11

**Authors:** Enrico Orsi, Pauline L. Folch, Vicente T. Monje-López, Bas M. Fernhout, Alessandro Turcato, Servé W. M. Kengen, Gerrit Eggink, Ruud A. Weusthuis

**Affiliations:** 1 Bioprocess Engineering, Department of Agrotechnology and Food, Wageningen University and Research, Wageningen, The Netherlands; 2 Laboratory of Microbiology, Department of Agrotechnology and Food, Wageningen University and Research, Wageningen, The Netherlands; 3 Biobased Products Food and Biobased Research, Wageningen University and Research, Wageningen, The Netherlands

**Keywords:** *Rhodobacter sphaeroides*, Amorphadiene, PHB, MEP, Mevalonate

## Abstract

*Rhodobacter sphaeroides* is a metabolically versatile bacterium capable of producing terpenes natively. Surprisingly, terpene biosynthesis in this species has always been investigated in complex media, with unknown compounds possibly acting as carbon and nitrogen sources. Here, a defined medium was adapted for *R. sphaeroides* dark heterotrophic growth, and was used to investigate the conversion of different organic substrates into the reporter terpene amorphadiene. The amorphadiene synthase was cloned in *R. sphaeroides*, allowing its biosynthesis via the native 2-methyl-d-erythritol-4-phosphate (MEP) pathway and, additionally, via a heterologous mevalonate one. The latter condition increased titers up to eightfold. Consequently, better yields and productivities to previously reported complex media cultivations were achieved. Productivity was further investigated under different cultivation conditions, including nitrogen and oxygen availability. This novel cultivation setup provided useful insight into the understanding of terpene biosynthesis in *R. sphaeroides*, allowing to better comprehend its dynamics and regulation during chemoheterotrophic cultivation.

## Introduction

The purple non-sulfur bacterium *Rhodobacter sphaeroides* is a metabolically versatile microorganism capable of growing, e.g., aerobically and anaerobically, photoautotrophically and heterotrophically [28, 43]. *R. sphaeroides* is therefore used as a model organism to study transcriptional regulation occurring during the transition from aerobic to photosynthetic metabolism [31, 34, 52]. In addition, its photosystem [32] and its quorum-sensing mechanism [37] have been investigated. More recently, industrial application of *R. sphaeroides* has been explored with respect to photoheterotrophic hydrogen biosynthesis [21] and chemoheterotrophic terpene production [5, 27].

Terpenes form the largest class of natural products with regard to their structural diversity and are synthesized by all organisms [11]. Interest for their application in the bio-economy is increasing. Currently they are used in the pharma and nutraceutical sectors [1, 20, 35, 39, 48], and they show potential for application also in the polymer industry [2] and as advanced biofuel [9, 53].

The structure of terpenes consists of a backbone of one or multiple isoprene units, with optional addition of other functional groups. Isoprene is synthesized via the mevalonate (MVA) pathway or the 2-methyl-d-erythritol-4-phosphate (MEP) pathway. The MVA pathway uses acetyl-CoA (Ac-CoA) and acetoacetyl-CoA (AA-CoA) as precursors, while the MEP pathway branches from glyceraldehyde-3-phosphate (GAP) and pyruvate. The MVA pathway is present in eukaryotes, most archaea and some Gram-positive bacteria, whereas all other prokaryotes use the MEP pathway. Only a few bacteria have both pathways [20]. Eventually, the two metabolic routes converge in the synthesis of the isoprene unit isopentenyl diphosphate (IPP) and its isomer dimethylallyl diphosphate (DMAPP). These two compounds are the building blocks for generating the different terpenes.

*Rhodobacter sphaeroides* is well known for its production of carotenoids and coenzyme Q10 (CoQ10). Both are synthesized via the MEP pathway, which is the native terpene biosynthetic route of *R. sphaeroides* [27]. Regulation of this pathway is known to occur through feedback inhibition by pyrophosphate compounds like IPP and DMAPP [3] and, at the transcriptional level, by oxygen tension [18, 49]. Various genetic engineering approaches have been performed on the MEP pathway of *R. sphaeroides* with the aim to increase the yield of CoQ10 [26, 54, 56]. The natural ability of *R. sphaeroides* to form terpenes has also been used for the production of aromatic terpenes for fragrances by expressing non-native terpene synthases, such as valencene synthase [5]. To increase the terpene production capacity the MVA pathway from Paracoccus zeaxanthinifaciens [14] has been introduced [29]. This microorganism was chosen because it is a member of the α-proteobacteria class, and therefore phylogenetically more closely related to *R. sphaeroides*.

To further improve terpene production in *R. sphaeroides*, thorough understanding of its physiology and regulation are required. All knowledge obtained so far regard this species is focused on CoQ10 production [26, 50], with the exception of lycopene [42]. Nevertheless, the current cultivation system has the following disadvantages: laborious extraction procedure for intracellular product determination (need of biomass collection and solvent based extraction), and low yields and titers of CoQ10 despite cultivation in rich media. It is, therefore, preferred to switch to a setup with defined medium and single carbon source. Moreover, the employment of a secreted reporter molecule would make product recovery and quantification easier.

In this work, adaptation of a defined Sistrom’s minimal medium [41] for dark heterotrophic growth and terpene production is presented. Moreover, the sesquiterpene amorpha-4,11-diene (amorphadiene from now on) was chosen as reporter molecule, allowing to obtain comparable yields to the literature employing rich medium for CoQ10 biosynthesis. Therefore, amorphadiene production was described in respect of growth and growth-limited condition induced by nutrients limitation. Two *R. sphaeroides* strains were used for this study. While one strain relies on IPP production exclusively via the native MEP pathway (MEP strain), the other additionally employs a heterologous MVA pathway (MEP + MVA strain). The results obtained contribute to improve the understanding of the versatile metabolism of *R. sphaeroides*. It also offers a new cultivation setup for further investigating the potential of its dark heterotrophic metabolism for biotechnological applications.

## Materials and methods

### Bacterial strains and plasmids

The strains and plasmids used are listed in Table 1. The strains and the plasmids not generated in this study were kindly provided by Isobionics BV.

**Table 1:** Bacterial strains and plasmids used in this study

Strains/plasmids	Characteristics	References
Strains		
MEP	Derivative of R. sphaeroides ATCC 35053 carrying pMEP. It expresses the gene amorphadiene synthase (ads) and supports terpene production via the native MEP pathway.	This study
MEP + MVA	Derivative of *R. sphaeroides* ATCC 35053 carrying pMVA. It expresses the gene amorphadiene synthase (ads) and supports terpene production via the native MEP pathway and the heterologous MVA pathway	[5, 29]
*E. coli* S17-1 (ATCC 47055)	Donor strain for di-parental conjugation	[40]
Plasmids		
pBBR1MCS-2	pBBR1 origin of replication, Km^R^, Mob site for mobilization during conjugation	[23]
pMEP	pBBR1MCS-2 + crtE promoter and *ads* (amorphadiene synthase)	This study
pMVA	pBBR1MCS-2 + PcrtE-*ads *+ MVA enzymes	[29]

### Plasmid construction

The pMEP plasmid was generated via restriction digestion. The amorphadiene synthase gene (*ads*) and its promoter PcrtE harboured in the pMVA plasmid were PCR amplified with overhangs for BamHI and SacI using the primers P17 (CAAAGAGCTCGGCGCGGGGCGCG) and P18 (ATTTGGATCCTTATCAGATCGACATGGGGTACACGAGC). The PCR amplicon and an empty pBBR1MCS-2 plasmid were digested with the two restriction enzymes and ligated in competent *Escherichia coli* TOP10 cells. Ligation was confirmed by colony PCR and sequencing. The isolated plasmid was conjugated in *R. sphaeroides* via di-parental conjugation mediated by *E. coli* S17-1 (ATC 47055). Successful conjugants were isolated, and the plasmid was sequenced. The primers used for sequencing were: P47: ATGTGCTGCAAGGCGATT; P48: TGACCGAGGAAAAGCCGAT; P49: AACTATAAGGACAAGAACGG; P50: TCGAGTGCTATTTCTGGGG; P51: AGGAGTGCTTCCGCTCCT; P52: GTTGAACAGGTCCGTGCG.

### Growth conditions

Cultivation of *R. sphaeroides* on defined minimal medium was performed using a modified Sistrom’s minimal medium (SMM) containing, if not differently specified: 3 or 10 g/L glucose, 3.48 g/L KH_2_PO_4_, 0.5 g/L NH_4_Cl, 0.1 g/L glutamic acid, 0.04 g/L l-aspartic acid, 0.5 g/L NaCl, 0.02 g/L nitrilotriacetic acid, 0.3 g/L MgSO_4_·7H_2_O, 0.00334 g/L CaCl_2_·2H_2_O, 0.002 g/L FeSO_4_·7H_2_O, and 0.0002 g/L (NH_4_)_6_Mo_7_O_24_. Trace elements were added 0.01% v/v from a stock solution containing: 17.65 g/L disodium EDTA, 109.5 g/L ZnSO_4_·7H_2_O, 50 g/L FeSO_4_·7H_2_O, 15.4 g/L MnSO_4_·7H_2_O, 3.92 g/L CuSO_4_·5H_2_O, 2.48 g/L Co(NO_3_)_2_·6H_2_O, and 0.114 g/L H_3_BO_3_. Vitamins were added 0.01% v/v from a stock containing: 10 g/L nicotinic acid, 5 g/L thiamine HCl, and 0.1 g/L biotin.

Preculturing of *R. sphaeroides* containing either pMEP or pMVA started by transferring the strains from frozen stock to Luria–Bertani (LB) agar plates supplied with kanamycin 50 µg/mL. Plates were incubated at 30 °C up to 72 h, until colonies were visible. Single colonies were passed from LB plates to 5 mL liquid LB containing 50 µg/mL kanamycin and incubated for 24 h at 30 °C with agitation rates of 250 rpm. Subsequently, the LB culture was conveyed in 250 or 300 mL Erlenmeyer flasks with 20% v/v of liquid Sistrom’s minimal medium (SMM) with 10 g/L glucose, 3.5 g/L NH_4_Cl and 50 µg/mL kanamycin. The precultures were incubated overnight at 30 °C, 250 rpm. Exponentially growing precultures were then harvested and, if necessary, washed twice with 9 g/L NaCl before being transferred to the cultivation medium.

### Screening for alternative carbon substrates

All cultivations were performed in a Kühner shaker incubator at 30 °C and with agitation rates of 250 rpm. *R. sphaeroides* strain MEP + MVA was grown on SMM using glucose as carbon source until mid-log phase. The cells were harvested by centrifugation at 8000 g for 10 min at room temperature, washed with 9 g/L NaCl and inoculated in SMM supplemented with the selected carbon source at a concentration (normalized for 10 g/L glucose) of 0.333 Cmol/L. Biological duplicates for each carbon source were grown in square bottom Applikon deep-well microtiter 24 wells polystyrene plates, incubated at 30 °C and agitation rates of 250 rpm. Growth was monitored over time by measuring the absorbance at OD_600_ using a Tecan Infinite M200 plate reader. In parallel, for amorphadiene detection, biological duplicates were inoculated in 100 mL shake flasks filled with 20 mL SMM with the selected carbon source and containing 10% v/v filter sterilized dodecane in the liquid phase.

### Shake flasks cultivation

Cultivation in shake flasks was performed in 250 mL Erlenmeyer flasks filled with 50 mL of SMM medium and filter sterilized dodecane (10% v/v of the liquid phase). The cultures were inoculated with a starting OD_600_ of 0.1 from exponentially growing precultures on SMM medium. The flasks were incubated as biological duplicates at 30 °C under an agitation of 250 rpm. Growth was monitored by measuring the absorbance at 600 nm. For assessment of nitrogen limitation on production, SMM medium with different initial NH_4_Cl concentrations (0.25–1.0 g/L) was employed. Initial glucose concentration was set at 3 g/L. Amorphadiene was measured once glucose depleted, and the pellet composition was analysed using the Total Organic Carbon analyser (TOC-L, Shimadzu).

### Bioreactor cultivation

*Rhodobacter sphaeroides* containing either pMEP (MEP strain) or pMVA (MEP + MVA strain) were cultivated at 30 °C in 0.5-L MiniBio 500 reactors from Applikon, with a working volume of 400 mL and 10% v/v of dodecane as organic phase. The bioreactors were sparged with 5 mL/min of compressed technical air and a continuous stirring of 1000 rpm was applied.

The DOT was kept at 40% by automatically varying the ratio between technical air and N_2_. The pH was maintained at 7.0 by automatic addition of 2.5 M NaOH. The bioreactors were inoculated with a starting OD_600_ of 0.25 from precultures grown in 250 mL shaker flasks containing 50 mL medium and 3.5 g/L NH_4_Cl. Samples of 2–15 mL were regularly taken to determine cell density and composition, substrate consumption and product formation.

### Effect of aeration on terpene biosynthesis

The effect of aeration on terpene biosynthesis was assessed by calculating amorphadiene yield on glucose (mg/g) after 48 h cultivation of 250 mL Erlenmeyer flasks containing different medium volumes. The medium used was SMM with 3 g/L of glucose and 1 g/L of NH_4_Cl (starting C/N in the medium below 5.5), which allowed an extended exponential growth. Preculturing of the MEP and MEP + MVA strains followed the protocol described in the correspondent section. Exponentially growing precultures were inoculated with a starting OD_600_ of 0.1 in the following volumes of SMM (10% v/v of dodecane): 10, 25, 100, and 200 mL. At the end of the cultivation, the content of the flask was spun at 4255 g for 5 min; the dodecane layer and the supernatant were collected for amorphadiene and glucose detection, respectively.

### Analytical methods

The cell density was monitored by measuring the optical density (OD_600_). The amorphadiene concentration was determined by dissolving 5% or 10% of the dodecane collected from the cultivations in ethyl acetate containing 0.2 mM tetradecane as internal standard. Samples were analysed on GC-FID 7890A from Agilent using a RESTEK Rxi-5ms column 0.25 µm (5% diphenyl, 95% dimethylpolysiloxane). Since amorphadiene is not commercially available, its isomer valencene (80% pure, kindly provided by Isobionics BV) was used as standard for calibration. The glucose and organic acid concentrations were determined using an Agilent 1290 Infinity (U)HPLC equipped with a guard column (Security Guard Cartridge System, Phenomenex). The compounds were separated on an organic acid column (Rezex ROA–Organic acid H^+^ 8% column, Phenomenex) at 55 °C with a flow of 0.5 mL/min 0.005 M H_2_SO_4_ as eluent. Alternatively, glucose was also measured with YSI 2950 from Shimadzu.

PHB was determined by centrifuging 10 mL culture at 4255 g for 20 min at room temperature. The supernatant was discarded and the cells were washed and centrifuged twice with MilliQ water, and not with NaCl 9 g/L for not interfering with the dry weight determination. Finally, the pellet was dried at 100 °C. The dried pellet was treated as described previously [6] and the PHB concentration was determined as crotonic acid using an Agilent 1290 Infinity (U)HPLC, using the same guard and elution column as for the glucose and organic acids determination.

The organic carbon and nitrogen content in pellet and supernatant was measured as non-purgeable organic carbon (NPOC) and as total nitrogen (TN) using a total organic carbon analyser (TOC-L) from Shimadzu. The nitrogen content measured in the pellet was used to calculate the amount of active biomass using the elemental composition of *R. sphaeroides* CH_1.99_O_0.5_N_0.19_, previously described in the literature for photoheterotrophic growth [46]. The C/N of the cells was calculated by dividing the NPOC and the TN values measured for the pellets.

## Results

### Amorphadiene as reporter molecule

The previous studies demonstrated that the best cultivation condition for terpene production in *R. sphaeroides* is heterotrophic dark cultivation [49] and this operational mode has been used ever since [19, 50, 51]. An attempt to summarize the yields and productivities already reported in the literature for this species is shown in Table 2. As can be observed, all the data focused on production of coenzyme Q10 (CoQ10, C_59_H_90_O_4_). Studying terpene biosynthesis using this molecule has several disadvantages, including the need of extraction for product quantification. Moreover, its complex structure and high molecular weight (863 g/mol) do not make it an ideal candidate for that purpose. Therefore, it was decided to engineer *R. sphaeroides* for producing the sesquiterpene amorpha-4,11-diene as reporter molecule. Such approach was already established in model organisms such as *E. coli* or *Saccharomyces cerevisiae* [30, 47]. This molecule (204 g/mol) is easily secreted by the microorganism and can be collected in an organic layer using a two-phase cultivation. *R. sphaeroides* was conjugated with a pBBR1MCS2 plasmid containing a heterologous MVA pathway derived from *Paracoccus zeaxanthinifaciens* [14, 29] expressing the gene for amorphadiene synthase (*ads*). From this plasmid, the *ads* gene under the control of the PcrtE promoter was amplified and inserted via restriction–ligation in an empty pBBR1MCS2 plasmid. Therefore, two *R. sphaeroides* strains were used, one synthesizing amorphadiene based only on the contribution of the native MEP pathway (MEP strain) and the other via co-expression of the MEP and constitutive MVA pathways (MEP + MVA strain). Product formation was confirmed for both strains via GC-FID determination.

**Table 2:** Comparative results of terpene yields and productions from various cultivation types using complex and defined media

Strain	Cultivation type	Yield (mol/mol glucose)	Production (mg/g CDW)	Medium	References
2.4.1 *wt*	Fed batch	0.00030	3.53	Complex	[54]
2.4.1 *ΔsdhB*		0.00039	4.59	Complex	
2.4.1 *wt*	Batch	0.00026	3.66	Complex	[56]
2.4.1 *RspPpsR*		0.00037	4.91	Complex	
2.4.1 *RspPE*		0.00038	5.67	Complex	
2.4.1 *RspMSC*	Batch	0.00024	3.48	Complex	[26]
2.4.1 *RspMegx*		0.0004	8.92	Complex	
2.4.1 *RspMQd*		0.00072	12.94	Complex	
KACC 91339P	Batch	*	6.34	Complex	[19]
	Fed Batch	*	8.12	Complex	
BCRC 13100	Batch	*	4.6	Complex	[51]
	Fed Batch	*	4.4	Complex	
BCRC 13100	Batch	0.0003	8.0	Complex	[49]
ATCC35053 derived MEP	Reactor—Batch	0.0006 ± 0.0001	1.41 ± 0.11	Defined	This study
ATCC35053 derived MEP + MVA		0.0051 ± 0.0014	10.99 ± 3.35	Defined	

All results presented with complex medium are associated to previous literature focused on Coenzyme Q10; instead, the data referred to defined medium was obtained using amorphadiene as reporter terpene

*Cannot be obtained since molasses was used as carbon source

### Selection of carbon source for heterotrophic terpene biosynthesis

Another relevant feature shown in Table 2 is the utilization of complex media for the biosynthesis of CoQ10. Such approach encumbers interpretation of substrates’ contribution to terpene biosynthesis. Nevertheless, Sistrom’s minimal medium (SMM) is a defined medium already employed for growing *R. sphaeroides*, although under photoheterotrophic conditions [41].

**Fig. 1:**
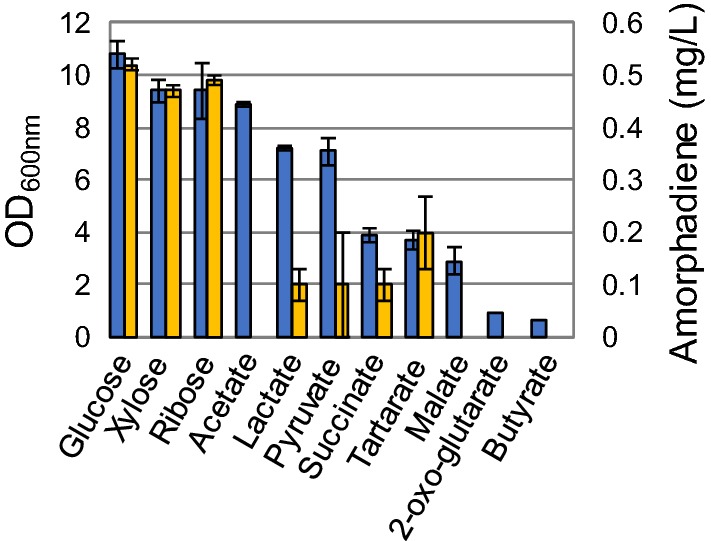
Screening of substrates for chemoheterotrophic amorphadiene biosynthesis in *Rhodobacter sphaeroides* (MEP + MVA strain) in modified Sistrom’s minimal medium using microtiter plates. OD at 600 nm (blue bars) and final amorphadiene titers (yellow bars) were used as parameters for the comparison. The initial concentration of the different substrates was normalized to 0.333 Cmol/L (color figure online)

We chose the same medium to assess its suitability for chemoheterotrophic growth using several carbon sources (at a concentration of 0.333 Cmol/L) as single substrates. The candidates chosen already proved to support growth under photoheterotrophic conditions [16], and can be relevant feedstocks for a biobased economy. As criteria for comparison, ‘final amorphadiene titre’ and ‘biomass concentration’ were used. The latter was expressed as maximal optical density (OD) measured at 600 nm. The outcome shows that sugars overall performed better than organic acids for supporting growth and terpene production (Fig. 1). While biomass concentrations and amorphadiene titres gave a similar outcome when sugars were used as substrates, a more pronounced variation was observed when organic acids were employed. The final ODs differed significantly, and not all acids supported amorphadiene synthesis. For instance, acetate resulted in a comparable biomass concentration as the sugars, but did not lead to any amorphadiene production. Succinate and malate resulted in similar microbial biomass concentrations, but the latter did not result in amorphadiene production. Glucose resulted in the highest biomass and terpene production, and was, therefore, chosen for further research.

### Growth and amorphadiene production in shake flasks

**Fig. 2:**
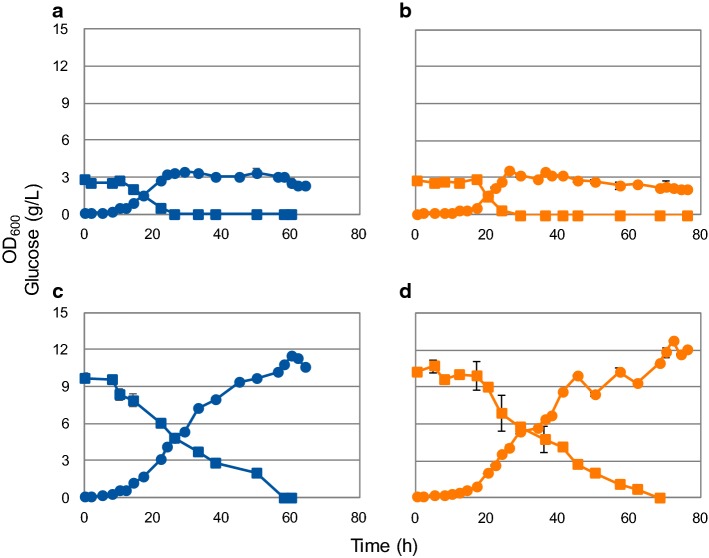
OD_600_ and glucose profiles of 250 mL shake flasks cultivations of *Rhodobacter sphaeroides* MEP (**a**, **c**) and MEP + MVA strains (**b**, **d**) grown at the initial concentration of 3 g/L (**a**, **b**) or 10 g/L glucose (**c**, **d**). Circles: OD at 600 nm; squares: glucose (g/L) (color figure online)

Further characterization of *R. sphaeroides* continued in shake flasks, where the behavior of MEP and MEP + MVA strains was compared. Growth was monitored over time (Fig. 2), and amorphadiene production was determined at the end of the cultivation. Two different initial glucose concentrations were tested: 3 g/L and 10 g/L. The two strains revealed a similar growth rate of 0.12 ± 0.01 h^−1^ at both sugar concentrations. After a short exponential phase, increase in absorbance continued in a rather linear mode until glucose depletion. This was observed best at the higher glucose concentration (Fig. 2c, d).

Amorphadiene titres amounted to similar levels for both glucose concentrations in the MEP strain. Their values were 1.8 ± 0.1 and 2.2 ± 0.2 mg/L for the 3 g/L and 10 g/L concentrations, respectively. For the MEP + MVA strain the difference between the two conditions was bigger. A 35% higher titre was measured in the culture with 10 g/L initial glucose concentration (30 ± 2 mg/L), compared to the 3 g/L initial glucose cultivation (22 ± 4 mg/L). The yield (mg amorphadiene−/−g glucose) was 12-fold higher and 13.5-fold higher for the MEP + MVA strain at 3 g/L and 10 g/L of glucose, respectively.

### Aerobic growth and production of amorphadiene and PHB in a reactor

**Fig. 3:**
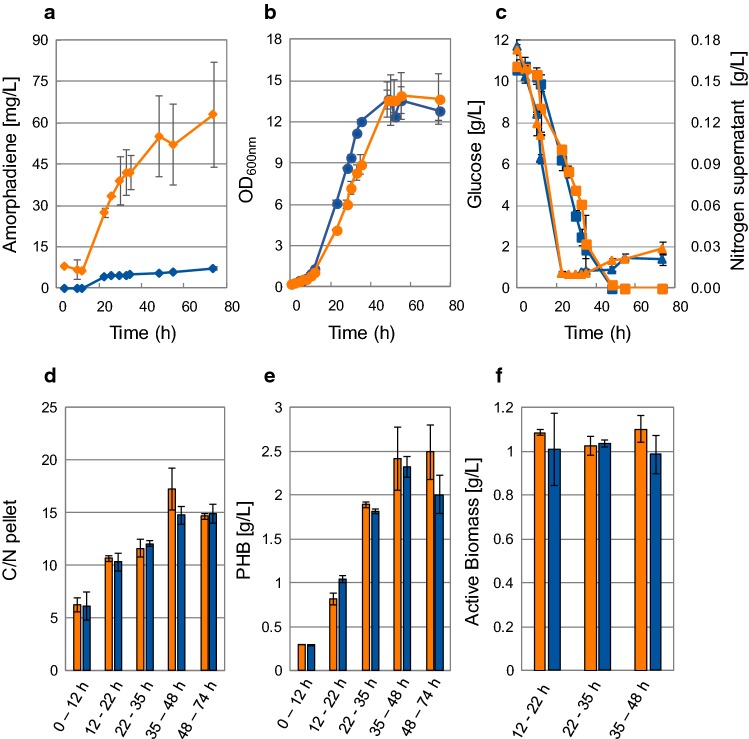
Heterotrophic cultivation of *Rhodobacter sphaeroides* MEP and MEP + MVA strains in 500-mL bioreactor. **a** Amorphadiene profile (diamonds) over time (mg/L). **b** OD_600_ profile (spheres). **c** Concentrations profiles (g/L) in the supernatant of glucose (squares) and nitrogen (triangles). **d** Pellet C/N and **e** intracellular PHB (g/L) during different time intervals within the batch cultivation. **f** Active biomass concentrations (g/L) during three different time intervals within the linear increase of OD_600_. Blue: MEP strain; orange: MEP + MVA strain (color figure online)

The linear growth observed (Fig. 2c, d) could be consequence of nutrient deficiency. In shake flasks oxygen is known to be a limiting nutrient [12]. Moreover, low oxygen tension is known to be a major effector of important metabolic switches in *R. sphaeroides* [52]. To allow enough oxygen transfer to the cultures, the batch cultivation was repeated in a 500-mL bioreactor, where the dissolved oxygen tension (DO) was kept constant at 40% air saturation (Fig. 3). Both the MEP and MEP + MVA strains were cultivated in SMM with 10 g/L of glucose, with 10% v/v dodecane as organic phase.

Amorphadiene production increased over time, with a tenfold difference measured between the two strains at the end of the cultivation (Fig. 3a). The yield on glucose calculated at the end of the fermentation is included in Table 2 for both strains. Analysis of the fermentation broth by HPLC showed that, except for glucose, no other organic compounds were present. In addition, in this case, a linear increase in OD was observed after a short exponential phase (*µ*_max_ MEP + MVA: 0.123 ± 0.002 h^−1^ and *µ*_max_ MEP: 0.136 ± 0.006 h^−1^). As the DO was maintained at 40% air saturation, linear growth could not have been caused by oxygen limitation. Evaluation of the medium composition indicated that nitrogen limitation could have occurred during the fermentation. N-limitation can be circumvented by fixing molecular N_2_ and *R. sphaeroides* is known to fix N_2_ via a nitrogenase complex [46]. Activity of the nitrogenase complex is extremely sensitive to oxygen [8]. Therefore, no N_2_ fixation was expected by *R. sphaeroides* under this experimental condition. To confirm a possible nitrogen limitation, the culture supernatant was analysed for total nitrogen (TN, via TOC-L). Moreover, the cell pellet was analysed for non-purgeable organic carbon (NPOC) and TN (both via TOC-L).

The switch from exponential to linear growth observed at 22 h (Fig. 3b) coincided with a depletion of nitrogen in the medium (Fig. 3c). This switch triggered the increase of the C/N ratio of the pellet over time, suggesting accumulation of a storage compound (Fig. 3d). The increase of the C/N ratio of the pellet was constant through the whole linear phase and levelled off at the stationary phase. Phototrophically grown *R. sphaeroides* is known to accumulate poly-β-hydroxybutyrate (PHB) when cultivated under nitrogen-limiting conditions [15, 46]. Accumulation of PHB under the applied heterotrophic growth conditions could explain the change in C/N ratio of the pellet. PHB accumulation could indeed be detected (Fig. 3e). Its accumulation stopped when the glucose in the medium was depleted, and the stationary phase was reached. From the TN measured in the pellet, it was possible to calculate the amount of active biomass (Fig. 3f) assuming the elemental composition of *R. sphaeroides* without PHB as CH_1.99_O_0.5_N_0.19_, which corresponds to a C/N of 5.3 [46]. The amount of active biomass did not increase during the linear growth phase and remained stable at about 1 g/L for both strains. Knowledge of the active biomass concentration allowed to calculate the specific productivity at the end of the fermentation, which resulted in about eightfold increase comparing MEP to MEP + MVA strain (Table 2).

### Growth-phase-dependent changes in PHB and amorphadiene

**Fig. 4:**
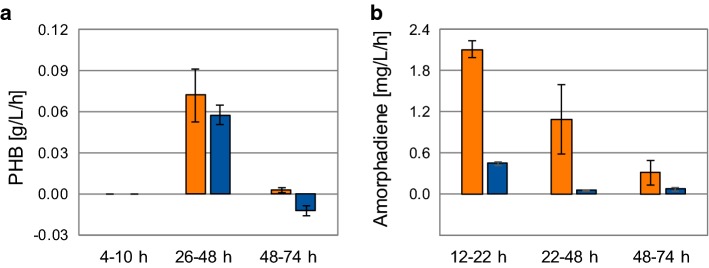
Volumetric rates for **a** PHB and **b** amorphadiene production calculated for the exponential, linear and stationary phase during chemoheterotrophic batch cultivation of *Rhodobacter sphaeroide*s in a 500-mL bioreactor using defined medium. Orange: MEP + MVA strain, blue: MEP strain (color figure online)

To better comprehend the observed changes in PHB and amorphadiene levels, we studied their production rates for each growth phase (Fig. 4): exponential (up to 22 h), linear (22–48 h), and stationary (48–74 h). Both strains showed a similar rate of PHB accumulation, which was highest during the linear growth phase (Fig. 4a). During the stationary phase, PHB was hardly produced, or even consumed. The rates of amorphadiene biosynthesis differed significantly between the two strains (Fig. 4b). For both, the value was highest during the exponential phase, and—as observed before—was significantly higher for the MEP + MVA strain. Once PHB accumulation started, amorphadiene biosynthesis stopped in the MEP strain. On the contrary, the MEP + MVA strain continued to produce amorphadiene, albeit lower when compared to the exponential phase. During the stationary phase, the MEP + MVA strain still produced amorphadiene, whereas production was negligible in the MEP strain. Interestingly, the fold difference in rates between the two strains was consistent during the exponential and stationary phases (fivefold higher for MEP + MVA strain). Nevertheless, during the PHB accumulation phase, the MEP + MVA strain’s rate was 20-fold higher compared to the one of the MEP strain. This suggests that the biggest difference in production between the two strains occurs during the accumulation of the storage compound.

### Effect of nitrogen limitation on cells’ productivity

**Fig. 5:**
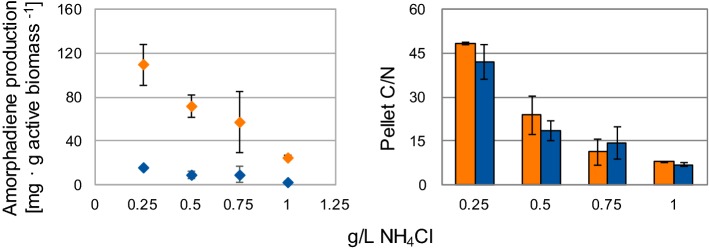
Effect of nitrogen limitation on amorphadiene production in *Rhodobacter sphaeroides*. Cultivation was performed in 250 mL shake flasks containing SMM with 3 g/L of glucose as substrate and different initial NH_4_Cl concentrations. Amorphadiene was measured from the organic layer once glucose depleted in the spent medium. Orange diamonds: MEP + MVA strain, blue diamonds: MEP strain (color figure online)

Volumetrically, amorphadiene biosynthesis seemed growth-associated. Nevertheless, growth-independent production of biotechnological compounds is an interesting approach, since less substrate would be consumed for biomass formation, allowing higher productivity per cell [25]. To assess the potential as growth-independent production platforms, the MEP and MEP + MVA strains were cultivated with different initial medium C/N. Amorphadiene productivity per grams of biomass was calculated at glucose depletion (Fig. 5a). Cells C/N was measured from the pellets at the end of the cultivation to confirm the different gradient of nutrient limitation (Fig. 5b). For both strains, the lowest NH_4_Cl concentration (highest cells C/N) corresponded to the highest specific production (MEP: 15.6 ± 0.1 mg/g biomass; MEP + MVA: 109.6 ± 18.9 mg/g biomass). These values correspond, respectively, to a 5.6-fold and 4.4-fold increase compared to the condition with the highest NH_4_Cl concentration (1 g/L, no nutrient limitation). Co-expression of MEP and MVA pathways in the MEP + MVA strain increased production during nutrient limitation, resulting in about sevenfold higher yield on biomass compared to the MEP strain at 0.25 g/L NH_4_Cl.

### Effect of aeration on terpene yields

Experimental evidence excluded a role of oxygen in determining the aforementioned transition from exponential to linear growth (Figs. 2, 3b). Nevertheless, oxygen tension is known to trigger important metabolic and morphologic changes in *R. sphaeroides* [17, 52]. In fact, a drop in the aeration determines biosynthesis of photosynthetic machineries, bacteriochlorophyll and carotenoids [33]. Using a defined medium could allow to determine the effect of aeration on terpene yields and assess its regulatory effect when expressing an additional heterologous MVA pathway. Cultivations of MEP and MEP + MVA strains have been performed with different volumes of SMM medium. The volume of medium in the flask affects the size of surface available for oxygen exchange between liquid and gas phases. Therefore, a relatively high and low oxygen level was obtained by applying, respectively, a high and low headspace/liquid ratio in the flasks [22].

**Fig. 6:**
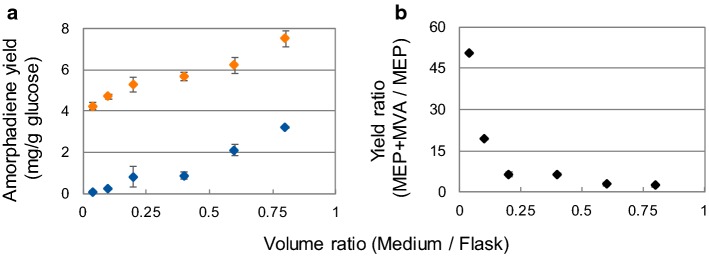
Effect of aeration on amorphadiene yield. *Rhodobacter sphaeroides* was cultivated with increasing volumes of liquid Sistrom’s minimal medium in 250 mL shake flasks containing 1 g/L NH_4_Cl and 3 g/L glucose. **a** Correlation between liquid volume ratio in the flasks and yield of amorphadiene on glucose for the MEP (blue diamonds) and MEP + MVA (orange diamonds) strains. **b** Relationship between the liquid volume ratio in the flasks and the yield ratio between the two strains (color figure online)

The SMM employed contained 3 g/L of glucose and 1 g/L of NH_4_Cl. This initial C/N ratio in the medium of 4.6 would allow complete exponential growth until glucose depletion, therefore, not interfering with the primary metabolism because of PHB accumulation. Each strain yielded the highest amount of amorphadiene when the ratio between medium and the working volume of the flask was the highest (0.8 v/v). Both strains showed a pronounced yield improvement with the increase of media volume over total flask volume (Fig. 6a). Comparison of the yields between the two strains showed that the biggest difference occurred at the highest headspace/liquid ratio (Fig. 6b). In fact, the MEP + MVA strain gave an almost 50-fold higher yield than the MEP strain. This difference in yield gradually decreased with the increase of medium volume in the flask. Interestingly, also the fold changes in yields within the same strain were different. For the MEP strain changing from maximal to minimal headspace/liquid ratio resulted in almost 40-fold yield increase, while for the MEP + MVA the change was barely twofold.

## Discussion

The biobased economy can benefit from the exploitation of industrial microorganisms for the production of economically relevant molecules [24]. Terpenes are among this type of compounds. Because of its versatile metabolism, the natural terpenoid producer *R. sphaeroides* offers an interesting platform for studying chemoheterotrophic production of these compounds and their regulation. Surprisingly, no information was available yet about chemoheterotrophic terpenoid biosynthesis in this bacterium employing defined medium. Here, a defined cultivation method was developed for identifying substrate-to-product conversions during terpene biosynthesis. Moreover, by expressing the amorphadiene synthase (*ads*) gene, it was possible to select an easily detectable reporter terpene via a two-phase cultivation system. Eventually, biosynthesis of amorphadiene on SMM allowed to reach comparable or even higher yields (mol/mol) to the ones reported for CoQ10 synthesized via complex medium (Table 2). Therefore, the proposed setup allowed to further characterize the physiological behavior associated with terpene biosynthesis in *R. sphaeroides* while using a single substrate.

An initial screening was performed to determine the optimal substrate supporting chemoheterotrophic growth and amorphadiene production in *R. sphaeroides*. The outcome revealed that consumption of sugars resulted in higher amorphadiene titres compared to organic acids. In particular, glucose resulted in the highest amorphadiene titre. Because measurement of the biomass concentration as OD_600_ was affected by nitrogen limitation, interpretation of effect of the substrate on growth can be biased. Since glucose catabolism revealed to be associated with the accumulation of a storage compound, a similar interpretation can in principle be extended to consumption of other substrates.

Amorphadiene biosynthesis in the MEP strain was clearly growth-associated, since production occurred only during biomass formation. This result was coherent with what was previously shown for *R. sphaeroides* producing CoQ10 [49]. Exponential growth resulted in the highest rate of amorphadiene biosynthesis also for the MEP + MVA pathway. Nevertheless, growth inhibition caused by nutrient limitation resulted in no amorphadiene production in the MEP strain. On the contrary, it allowed terpene biosynthesis in the MEP + MVA strain while PHB was stored, reaching an eightfold increase in titer and productivity. PHB accumulation has already been described as major by-product also during photoheterotrophic H_2_ fermentation as consequence of nitrogen limitation [38, 44]. Both PHB and MVA pathways share acetoacetyl-CoA as committed precursor. Such proximity between the two metabolic routes and constitutive expression of the MVA enzymes could explain the persistence of amorphadiene production also during growth limitation.

Incremented intracellular PHB resulted in increase of pellet C/N, and resulted in a linear raise in OD_600_. The absence of other organic compounds detected via HPLC implies that PHB was the major by-product of the process. Accumulation of this storage compound is known to function as carbon sink and as NADPH oxidation option during nutrient limitation [13]. NADP^+^ regeneration via PHB formation could help in providing a sink for reducing equivalent to support glucose catabolism when biomass accumulation is prevented. Two pathways can reduce NADP^+^ during glucose catabolism. These are the pentose phosphate pathway and the Entner-Doudoroff (ED) pathway. The latter is known to be the main glycolytic pathway employed by *R. sphaeroides* [10]. Therefore, it is suggested that PHB formation could be exploited as carbon sink and cofactor regeneration pathway for supporting glucose catabolism via the ED pathway during nutrient limitation. Such hypothesis could benefit from cultivation with ^13^C substrates for quantifying glycolytic pathways contribution under these conditions. Nevertheless, connection between the reducing equivalents of ED and PHB pathways was already proven in *E.coli* [13]. Additional supporting evidence of their association was that the overexpression of the NADP(H)-dependent enzymes of the two pathways resulted in increased PHB accumulation in the same species [7].

Although the highest volumetric rate of amorphadiene production occurred during exponential growth (Fig. 4b), its molecular formula (C_15_H_24_) suggests that its biosynthesis could in principle increase during nitrogen-limited conditions [25]. In such scenario, no carbon would be directed towards amino acid and protein biosynthesis, allowing a higher carbon flux towards amorphadiene, as previously suggested for its production in *E. coli* [45]. Consistently, cultivation of *R. sphaeroides* under nutrient limitation increased cells productivity (Fig. 5). Expression of a heterologous MVA pathway proved to support amorphadiene biosynthesis even further under these circumstances. Further research to increase terpene biosynthesis under growth-independent conditions (e.g. via rational re-design of *R. sphaeroides* metabolism) is, therefore, desirable.

Oxygen is known to be a major environmental factor regulating *R. sphaeroides* metabolism. Variations in oxygen tension affect the transcriptional regulation coordinated by three main systems: FnrL, PrrA/PrrB and PpsR [4, 52]. Among these candidates, PpsR is known to inhibit transcription of carotenoids and MEP-associated genes [36, 55]. By varying the volume of headspace transfer in the cultures, it was possible to qualitatively assess the effect of aeration on amorphadiene biosynthesis. The first piece of evidence was that both strains produced the highest amount of amorphadiene when the headspace volume was lowest. Nevertheless, presence of the MVA pathway allowed higher yields also when the headspace volume was maximal, suggesting a bypass of the native oxygen-based regulation affecting the native MEP pathway. By reducing the volume of the air in the flask, and accordingly the oxygen supply, the yield difference between the two strains decreased progressively. Accordingly, the MEP strain showed a higher yield-fold increase switching from maximal to minimal headspace volumes, while for the MEP + MVA strain this increase was moderate. MEP pathway expression is triggered by low oxygen tension [36], when it supports biosynthesis of photosystem-associated molecules such as carotenoids. Therefore, its activation can be expected under micro-aerobic conditions in both strains. Interestingly, the two strains showed a similar trend of variation in yield moving from maximal to minimal headspace volumes. This suggests that an improved yield in the MEP + MVA strain could be orchestrated by an increased contribution of the native MEP pathway. This speculation would benefit from analysis of the split ratio of the two terpene biosynthetic routes, e.g., by ^13^C-labeled cultivation. Furthermore, genetic engineering approaches could be used for deregulating the native MEP pathway, as already attempted for reducing competitive expression of carotenoids during coenzyme Q10 biosynthesis [55].

This work describes the implementation of a novel cultivation system for studying the heterotrophic metabolism of *R. sphaeroides* under defined culture conditions. Moreover, it was employed for investigating the dynamics of terpene biosynthesis in this species using different organic substrates and nutrients (nitrogen and oxygen) availabilities. When introducing a heterologous mevalonate pathway and amorphadiene synthase, the highest yields reported for this species were achieved. All in all, this study serves as basis for further improving the metabolism of *R. sphaeroides* for enhanced terpene biosynthesis.

## References

[1] Ajikumar PK, Tyo K, Carlsen S, Mucha O, Phon TH, Stephanopoulos G (2008). Terpenoids: opportunities for biosynthesis of natural product drugs using engineered microorganisms. Mol Pharm.

[2] Alvès MH, Sfeir H, Tranchant JF, Gombart E, Sagorin G, Caillol S, Billon L, Save M (2014). Terpene and dextran renewable resources for the synthesis of amphiphilic biopolymers. Biomacromol.

[3] Banerjee A, Wu Y, Banerjee R, Li Y, Yan H, Sharkey TD (2013). Feedback inhibition of deoxy-D-xylulose-5-phosphate synthase regulates the methylerythritol 4-phosphate pathway. J Biol Chem.

[4] Bauer CE, Setterdahl A, Wu J, Robinson BR (2009) Regulation of gene expression in response to oxygen tension. In: The purple phototrophic bacteria, pp 707–725

[5] Beekwilder J, van Houwelingen A, Cankar K, van Dijk ADJ, de Jong RM, Stoopen G, Bouwmeester H, Achkar J, Sonke T, Bosch D (2014). Valencene synthase from the heartwood of Nootka cypress (*Callitropsis nootkatensis*) for biotechnological production of valencene. Plant Biotechnol J.

[6] Carpine R, Raganati F, Olivieri G, Hellingwerf KJ, Pollio A, Salatino P, Marzocchella A (2018). Poly-β-hydroxybutyrate (PHB) production by *Synechocystis* PCC6803 from CO_2_: model development. Algal Res.

[7] Choi JC, Shin HD, Lee YH (2003). Modulation of 3-hydroxyvalerate molar fraction in poly(3-hydroxybutyrate-3-hydroxyvalerate) using *Ralstonia eutropha* transformant co-amplifying *phbC* and NADPH generation-related *zwf* genes. Enzyme Microb Technol.

[8] Dixon R, Kahn D (2004). Genetic regulation of biological nitrogen fixation. Nat Rev Microbiol.

[9] Fortman JL, Chhabra S, Mukhopadhyay A, Chou H, Lee TS, Steen E, Keasling JD (2008). Biofuel alternatives to ethanol: pumping the microbial well. Trends Biotechnol.

[10] Fuhrer T, Fischer E, Sauer U (2005). Experimental identification and quantification of glucose metabolism in seven bacterial species. J Bacteriol.

[11] Gershenzon J, Dudareva N (2007). The function of terpene natural products in the natural world. Nat Chem Biol.

[12] Gupta A, Rao G (2003). A study of oxygen transfer in shake flasks using a non-invasive oxygen sensor. Biotechnol Bioeng.

[13] Hong SH, Park SJ, Moon SY, Park JP, Lee SY (2003). In silico prediction and validation of the importance of the Entner-Doudoroff pathway in poly(3-hydroxybutyrate) production by metabolically engineered *Escherichia coli*. Biotechnol Bioeng.

[14] Hümbelin M, Thomas A, Lin J, Li J, Jore J, Berry A (2002). Genetics of isoprenoid biosynthesis in *Paracoccus zeaxanthinifaciens*. Gene.

[15] Hustede E, Steinbüchel A, Schlegel HG (1993). Relationship between the photoproduction of hydrogen and the accumulation of PHB in non-sulphur purple bacteria. Appl Microbiol Biotechnol.

[16] Imam S, Noguera DR, Donohue TJ (2013). Global insights into energetic and metabolic networks in *Rhodobacter sphaeroides*. BMC Syst Biol.

[17] Kaplan S, Eraso J, Roh JH (2005). Interacting regulatory networks in the facultative photosynthetic bacterium, *Rhodobacter sphaeroides* 2.4.1. Biochem Soc Trans.

[18] Khan NE, Nybo SE, Chappell J, Curtis WR (2015). Triterpene hydrocarbon production engineered into a metabolically versatile host- *Rhodobacter capsulatus*. Biotechnol Bioeng.

[19] Kien NB, Kong IS, Lee MG, Kim JK (2010). Coenzyme Q10 production in a 150-l reactor by a mutant strain of *Rhodobacter sphaeroides*. J Ind Microbiol Biotechnol.

[20] Kirby J, Keasling JD (2009). Biosynthesis of plant isoprenoids: perspectives for microbial engineering. Annu Rev Plant Biol.

[21] Koku H, Eroğlu I, Gündüz U, Yücel M (2002). Aspects of the metabolism of hydrogen production by *Rhodobacter sphaeroides*. Int J Hydrogen Energy.

[22] de Koning W, Weusthuis RA, Harder W, Dijkhuizen L (1990). Methanol-dependent production of dihydroxyacetone and glycerol by mutants of the methylotrophic yeast *Hansenula polymorpha* blocked in dihydroxyacetone kinase and glycerol kinase. Appl Microbiol Biotechnol.

[23] Kovach ME, Elzer PH, Hill DS, Robertson GT, Farris MA, Roop RM, Peterson KM (1995). Four new derivatives of the broad host range cloning vector PBBR1MCS, carrying different antibiotic resistance cassettes. Gene.

[24] Lee SY, Mattanovich D, Villaverde A (2012). Systems metabolic engineering, industrial biotechnology and microbial cell factories. Microb Cell Fact.

[25] Li S, Jendresen CB, Nielsen AT (2016). Increasing production yield of tyrosine and mevalonate through inhibition of biomass formation. Process Biochem.

[26] Lu W, Ye L, Lv X, Xie W, Gu J, Chen Z, Zhu Y, Li A, Yu H (2015). Identification and elimination of metabolic bottlenecks in the quinone modification pathway for enhanced coenzyme Q 10 production in *Rhodobacter sphaeroides*. Metab Eng.

[27] Lu W, Ye L, Xu H, Xie W, Gu J, Yu H (2014). Enhanced production of coenzyme Q10 by self-regulating the engineered MEP pathway in *Rhodobacter sphaeroides*. Biotechnol Bioeng.

[28] Madigan MT, Howard G (1979). Growth of the photosynthetic bacterium Rhodopseudomonas capsulata chemoautotrophically in darkness with H_2_ as the energy source. J Bacteriol.

[29] Hümbelin M, Beekwilder J, Kierkels JGT (2014) *Rhodobacter* for preparing terpenoids. Retrived from: https://patents.google.com/patent/WO2014014339A3/en. Patent No: WO2014014339A3

[30] Martin VJJ, Pitera DJ, Withers ST, Newman JD, Keasling JD (2003). Engineering a mevalonate pathway in Escherichia coli for production of terpenoids. Nat Biotechnol.

[31] Masuda S, Bauer CE (2002). AppA is a blue light photoreceptor that antirepresses photosynthesis gene expression in *Rhodobacter sphaeroides*. Cell.

[32] Moskvin OV, Gomelsky L, Gomelsky M (2005). Transcriptome analysis of the *Rhodobacter sphaeroides* PpsR regulon: PpsR as a master regulator of photosystem development. J Bacteriol.

[33] Niederman RA (2013). Membrane development in purple photosynthetic bacteria in response to alterations in light intensity and oxygen tension. Photosynth Res.

[34] O’Gara JP, Eraso JM, Kaplan S (1998). A redox-responsive pathway for aerobic regulation of photosynthesis gene expression in *Rhodobacter sphaeroides* 2.4.1. J Bacteriol.

[35] Paddon CJ, Keasling JD (2014). Semi-synthetic artemisinin: a model for the use of synthetic biology in pharmaceutical development. Nat Rev Microbiol.

[36] Pappas CT, Sram J, Moskvin OV, Ivanov PS, Mackenzie RC, Choudhary M, Land ML, Larimer FW, Kaplan S, Gomelsky M (2004). Construction and validation of the *Rhodobacter sphaeroides* 2.4.1 DNA microarray: transcriptome flexibility at diverse growth modes. J Bacteriol.

[37] Puskas A, Greenberg EP, Kaplan S, Schaefer AL (1997). A quorum-sensing system in the free-living photosynthetic bacterium *Rhodobacter sphaeroides*. J Bacteriol.

[38] Ryu M-H, Hull NC, Gomelsky M (2014). Metabolic engineering of *Rhodobacter sphaeroides* for improved hydrogen production. Int J Hydrogen Energy.

[39] Schempp FM, Drummond L, Buchhaupt M, Schrader J (2017). Microbial cell factories for the production of terpenoid flavor and fragrance compounds. J Agric Food Chem.

[40] Simon R, Priefer U, Pühler A (1983). A broad host range mobilization system for in vivo genetic engineering: transposon mutagenesis in Gram negative bacteria. Nat Biotechnol.

[41] Sistrom WR (1960). A Requirement for sodium in the growth of *Rhodopseudomonas spheroides*. J Gen Microbiol.

[42] Su A, Chi S, Li Y, Tan S, Qiang S, Chen Z, Meng Y (2018). Metabolic Redesign of Rhodobacter sphaeroides for Lycopene Production. J Agric Food Chem.

[43] Tabita RF (1995). The biochemistry and metabolic regulation of carbon metabolism and CO_2_ fixation in purple bacteria.

[44] Tao Y, Liu D, Yan X, Zhou Z, Lee JK, Yang C (2012). Network identification and flux quantification of glucose metabolism in *Rhodobacter sphaeroides* under photoheterotrophic H_2_-producing conditions. J Bacteriol.

[45] Tsuruta H, Paddon CJ, Eng D, Lenihan JR, Horning T, Anthony LC, Regentin R, Keasling JD, Renninger NS, Newman JD (2009). High-level production of amorpha-4, 11-diene, a precursor of the antimalarial agent artemisinin, in *Escherichia coli*. PLoS One.

[46] Waligórska M, Seifert K, Górecki K, Moritz M, Łaniecki M (2009). Kinetic model of hydrogen generation by *Rhodobacter sphaeroides* in the presence of NH4^+^ ions. J Appl Microbiol.

[47] Westfall PJ, Pitera DJ, Lenihan JR, Eng D, Woolard FX, Regentin R, Horning T, Tsuruta H, Melis DJ, Owens A, Fickes S, Diola D, Benjamin KR, Keasling JD, Leavell MD, McPhee DJ, Renninger NS, Newman JD, Paddon CJ (2012). Production of amorphadiene in yeast, and its conversion to dihydroartemisinic acid, precursor to the antimalarial agent artemisinin. Proc Natl Acad Sci USA.

[48] Withers ST, Keasling JD (2007). Biosynthesis and engineering of isoprenoid small molecules. Appl Microbiol Biotechnol.

[49] Yen H-W, Chiu C-H (2007). The influences of aerobic-dark and anaerobic-light cultivation on CoQ10 production by *Rhodobacter sphaeroides* in the submerged fermenter. Enzyme Microb Technol.

[50] Yen HW, Feng CY, Kang JL (2010). Cultivation of *Rhodobacter sphaeroides* in the stirred bioreactor with different feeding strategies for CoQ10 production. Appl Biochem Biotechnol.

[51] Yen HW, Shih TY (2009). Coenzyme Q10 production by *Rhodobacter sphaeroides* in stirred tank and in airlift bioreactor. Bioprocess Biosyst Eng.

[52] Zeilstra-Ryalls JH, Kaplan S (2004). Oxygen intervention in the regulation of gene expression: the photosynthetic bacterial paradigm. Cell Mol Life Sci.

[53] Zhang F, Cardayre DSB, Keasling JD (2012). Microbial engineering for the production of advanced biofuels. Nature.

[54] Zhang J, Gao D, Cai J, Liu H, Qi Z (2018). Improving coenzyme Q10 yield of *Rhodobacter sphaeroides* via modifying redox respiration chain. Biochem Eng J.

[55] Zhu Y, Lu W, Ye L, Chen Z, Hu W, Wang C, Chen J, Yu H (2017). Enhanced synthesis of Coenzyme Q10 by reducing the competitive production of carotenoids in *Rhodobacter sphaeroides*. Biochem Eng J.

[56] Zhu Y, Ye L, Chen Z, Hu W, Shi Y, Chen J, Wang C, Li Y, Li W, Yu H (2017). Synergic regulation of redox potential and oxygen uptake to enhance production of coenzyme Q10 in *Rhodobacter sphaeroides*. Enzyme Microb Technol.

